# Fast and Accurate Gamma Imaging System Calibration Based on Deep Denoising Networks and Self-Adaptive Data Clustering

**DOI:** 10.3390/s23052689

**Published:** 2023-03-01

**Authors:** Yihang Zhu, Zhenlei Lyu, Wenzhuo Lu, Yaqiang Liu, Tianyu Ma

**Affiliations:** 1 Department of Engineering Physics, Tsinghua University, Beijing 100084, China; 2 Key Laboratory of Particle & Radiation Imaging, Ministry of Education, Tsinghua University, Beijing 100084, China; 3 Institute for Precision Medicine, Tsinghua University, Beijing 100084, China

**Keywords:** experimental calibration, system matrix, deep learning, image denoising, gamma imager

## Abstract

Gamma imagers play a key role in both industrial and medical applications. Modern gamma imagers typically employ iterative reconstruction methods in which the system matrix (SM) is a key component to obtain high-quality images. An accurate SM could be acquired from an experimental calibration step with a point source across the FOV, but at a cost of long calibration time to suppress noise, posing challenges to real-world applications. In this work, we propose a time-efficient SM calibration approach for a 4π-view gamma imager with short-time measured SM and deep-learning-based denoising. The key steps include decomposing the SM into multiple detector response function (DRF) images, categorizing DRFs into multiple groups with a self-adaptive K-means clustering method to address sensitivity discrepancy, and independently training separate denoising deep networks for each DRF group. We investigate two denoising networks and compare them against a conventional Gaussian filtering method. The results demonstrate that the denoised SM with deep networks faithfully yields a comparable imaging performance with the long-time measured SM. The SM calibration time is reduced from 1.4 h to 8 min. We conclude that the proposed SM denoising approach is promising and effective in enhancing the productivity of the 4π-view gamma imager, and it is also generally applicable to other imaging systems that require an experimental calibration step.

## 1. Introduction

A gamma imager is an important tool in industrial and medical applications for visually inspecting and measuring the spatial distribution of gamma radiation. Industrial gamma imagers, such as coded-aperture gamma cameras [1,2] and Compton cameras [3,4], are widely used in homeland security and nuclear emergency response scenarios. Medical gamma imaging devices, such as planar gamma cameras, Single Photon Emission Computed Tomography (SPECT), and Positron Emission Computed Tomography (PET), have been the backbone of clinical and preclinical molecular imaging.

In either of the above gamma imagers, image reconstruction is a key step that solves source distribution images from the measured photon position and energy information. Most modern gamma imagers employ a statistical image reconstruction algorithm, such as the maximum likelihood expectation maximization (MLEM) algorithm [5]. Recent studies have suggested that the iterative reconstruction methods could yield better image resolution and signal-to-noise performance [4,6] compared to analytical methods such as back-projection, filtered back-projection, and correlation analysis methods [7].

One of the recognized merits of iterative reconstruction is its incorporation of an accurate response of geometrical, physical, and instrumental factors of the imaging device in the reconstruction process, represented in the form of a system matrix (SM). An accurate SM has been proven to be critical for improving resolution, quantitative accuracy, and reducing the noise for gamma imaging systems [8,9,10], and inaccurate SM may degrade image quality or even introduce artifacts [11]. Therefore, accurate SM calibration is of vital importance for gamma imaging instruments.

Common SM generation methods include analytical calculation [12,13,14], Monte Carlo simulation [15,16,17], and experimental measurement [18,19,20,21,22]. Analytical calculation is fast but not applicable to systems with complex geometries. Monte Carlo simulation enables more accurate system modeling but requires powerful computational capabilities. Moreover, the discrepancies between the actual properties of detector crystals and digital processing elements can hardly be considered in both analytical calculation and Monte Carlo simulation, making the SM inaccurate when either calculated or simulated.

In comparison, experimental measurement of SM is proven to be the most accurate approach, which directly incorporates instrumental factors in the acquisition of SM. This method has been applied in various imaging systems, such as SPECT [19,20,21], PET [23], and industrial gamma cameras [22,24]. However, a significant drawback of the experimental SM calibration approach is the time-consuming measurement process [25]. Typically, this approach requires precisely moving a small-sized point source to acquire system response across the image field-of-view (FOV). Acquiring enough counts for every pair of image voxels and detector bins is typically a long and laborious process. Simply reducing the measuring time would inevitably induce statistical noise.

In our lab, we have developed a high-sensitivity 4π-view gamma imager with a novel collimator-less design [26]. We have demonstrated that with a 3D position-sensitive scintillation detector, photon attenuation in one detector bin induces a direction-dependent response in the other detector bins, which provides sufficient information for reconstructing the gamma radiation image in the 4π-FOV [26,27,28,29]. In this system, we use a point source to calibrate the SM in real experiments. However, when the designed gamma imager undergoes a volume production process, there is a strong need to speed up the calibration procedure without trading calibration accuracy.

This work aims to develop an efficient SM calibration method in an experiment. We propose to denoise the SM measured in a short time duration. Traditional noise suppression methods include Gaussian filtering [30], non-local means filtering [31], and block-matching 3D filtering [32], but they are not universally suitable for complex images with many details. In recent years, various deep learning methods have been proposed, especially in medical imaging fields. Endeavors have been made to apply denoising networks on the sinogram domain [33,34], during image post-processing [35,36], or embed them in a reconstruction framework [37,38]. Among them, an encoder–decoder U-net model structure [39] is broadly used. The encoder of U-net captures and analyzes the context of the input image while the decoder enables precise localization as well as generates the output image. The skip connections between them propagate low-level features to high-resolution layers and compensate for information loss. Therefore, U-net can extract and preserve the image features of different levels, helping to recover details of medical images. Additionally, existing works show that U-net-based methods have promising performance in Poisson noise suppression [33,34,40], which is consistent with the task in SM denoising.

In this work, we propose to realize the fast calibration of SM through deep-learning-based denoising of fast-calibrated SM. We investigated two deep neural networks, one with a U-net architecture and the other with a residual U-net structure. We evaluated the accuracy of denoised SM through comparisons with a Gaussian-filtered SM in terms of the structural similarity index (SSIM) between the denoised SM and a long-time measured one. We also investigated the gamma positioning accuracy and image resolution performance of the gamma images with the denoised SM.

## 2. Materials and Methods

### 2.1. The 4π-View Gamma Imager

The proposed SM denoising approach is validated with the 4π-view gamma imager developed in our lab. To facilitate understanding, we briefly describe the gamma imager design in this section. Readers are referred to Ref. [26] for more details.

**Figure 1:**
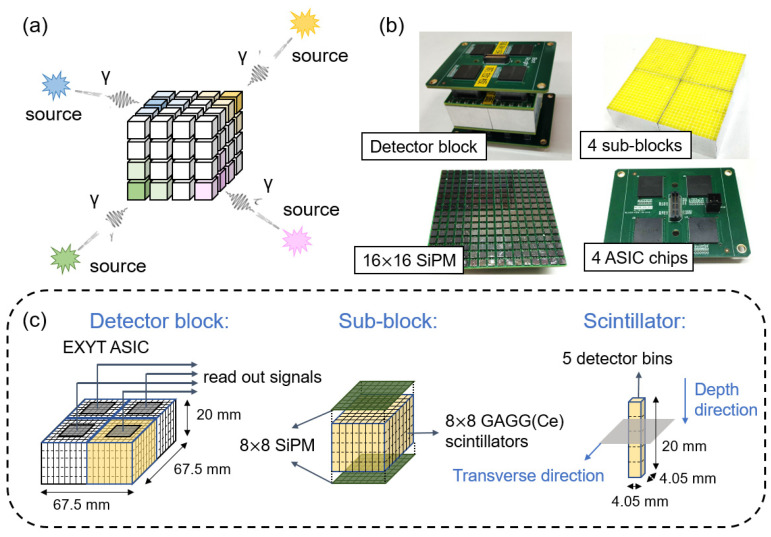
(**a**) Illustration of imaging concept of 3D position-sensitive detector block. (**b**) Pictures of detector block prototype. (**c**) Illustration of the detector block design.

As shown in Figure 1a, the core component of the gamma imager is a 3D position-sensitive radiation detector block. When gamma photons emitted from surrounding radiation source(s) hit the detector block, every detector element has a different photon detection probability depending on the direction of the gamma ray due to varied photon attenuation from other detector elements on the photon path. Therefore, the accumulated photon events’ distribution over a period of time reflects the directional distribution of radiation sources.

As shown in Figure 1b,c, we assembled a realistic detector block with cerium-doped gadolinium aluminum gallium garnet (GAGG(Ce)) scintillator and silicon photomultiplier (SiPM) arrays on both ends. The entire scintillator block was 67.5 × 67.5 × 20 mm^3^ in size, consisting of 16 × 16 GAGG(Ce) scintillators (EPIC Crystal, Shanghai, China) with a size of 4.05 × 4.05 × 20 mm^3^. Each scintillator was smeared with a totally reflective material, BaSO_4_ (EPIC Crystal, Shanghai, China), on the four side surfaces. Two 16 × 16 SiPM arrays (FJ30035, Onsemi, Phoenix, AZ, USA) were coupled to the dual end surfaces of the scintillator block.

The signals of SiPM arrays were read by a lab-developed ASIC chip [41]. Since each ASIC had 8 × 8 channels, we virtually divided the detector block into 2 × 2 sub-blocks (Figure 1b,c), and each sub-block contained 8 × 8 GAGG(Ce) scintillators. One should note that the sub-block division strategy impacts the detector performance as well as the denoising process (see Section 2.3 and Section 2.4 for details).

**Figure 2:**
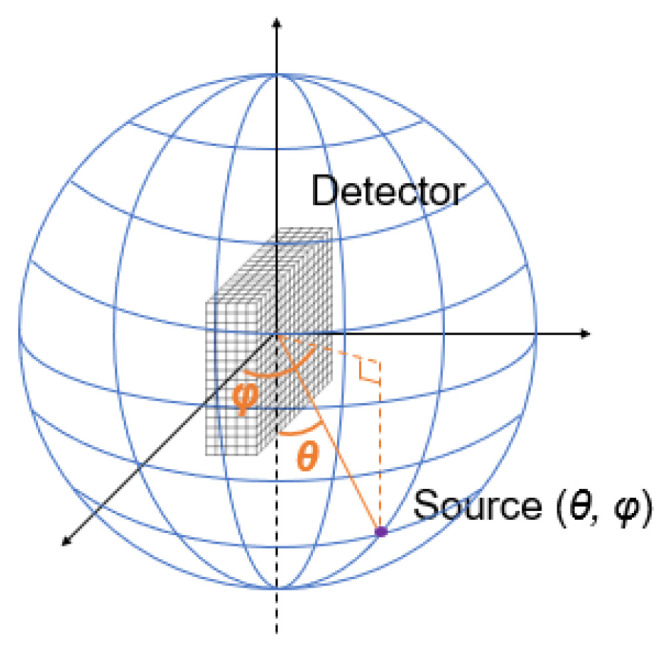
Illustration of the geometrical setup of the imaging problem.

To formulate the imaging problem, we defined a spherical coordinate system with the origin at the geometrical center of the detector. As shown in Figure 2, the image was defined on a 4π-view sphere surface, denoted by the polar angle *θ* and azimuth angle *φ*. We discretized the image domain into 181(*θ*) × 360(*φ*) pixels with a pixel size of 1° × 1°. The radioactivity in the *i*-th image pixel is denoted as $x_{i}$.

The detector block measures the 3D position for each detected photon and histograms the measured events into projection {$p_{j}$}, where $p_{j}$ denotes the number of photon events in the *j*-th detector bin. In the transverse direction, the intrinsic detector resolution is determined by the scintillator size; therefore, there are 16 × 16 detector bins transversely. In the depth direction, we use a dual-end read-out technique [27] to calculate the photon interaction position. According to our measurement, the position estimation accuracy is ~4 mm, resulting in 5 detector bins in the depth direction. Therefore, in each scintillator block, we define a total of 16 × 16 × 5 detector bins.

The imaging task aims to reconstruct the radioactivity image $\left\{x_{i}\right\}$ using the measured projection dataset $\left\{p_{j}\right\}$. We used the MLEM algorithm [5] in image reconstruction as follows:

$$x_{i}^{k+1}=\frac{x_{i}^{k}}{\sum_{j}a_{ij}}\underset{j}{\sum }a_{ij}\frac{p_{j}}{\sum_{i}x_{i}^{k}a_{ij}}$$

where $x_{i}^{k+1}$ and $x_{i}^{k}$ indicate the reconstructed images after *k* + 1 and *k* iterations, respectively. The system matrix {$a_{ij}$} denotes the probability of one photon emitted from the *i*-th image voxel and being detected in the *j*-th detector bin.

### 2.2. System Matrix Calibration and Detector Response Function

**Figure 3:**
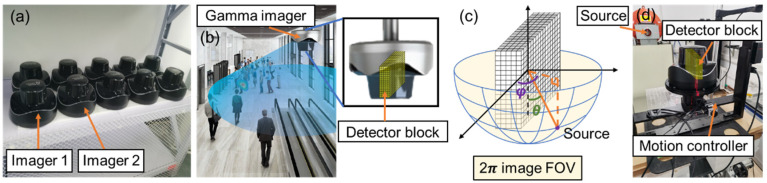
(**a**) Picture of several gamma imagers. (**b**) Illustration of radiation source monitoring scenario. (**c**) Illustration of system matrix calibration setup. (**d**) Picture of realistic system matrix calibration process.

Figure 3a demonstrates a realistic scheme, with multiple gamma imagers manufactured in a volume-producing procedure, that requires extensive SM calibrations. Due to hardware and assembling-induced variations, the SM of each imager needs to be individually calibrated. In this study, we chose two representative imagers, Imager 1 and Imager 2.

In the radiation source monitoring application scenario illustrated in Figure 3b, the gamma imager is installed on the ceiling with a top-view posture. In this case, a 2π image FOV is required. Therefore, although the gamma imager itself allows 4π-imaging, in what follows, we define a 2π-FOV for the realistic imaging system (Figure 3c). The developed methodology is expected to be applicable in the entire 4π-FOV.

In the experimental calibration process, the SM is measured with a point source surrounding the imager and then pre-stored in the computer. Each imager is mounted on a motion controller so that it is rotatable about the center and is exposed to a stationary radioactive source (Figure 3d) to calibrate the system matrix. We rotate the imager to allow hemispherical coverage of the illumination of the point source, which makes *θ* from 0° to 90° and *φ* from 0° to 359° (Figure 3c). The whole calibration process is described as follows:

(1) Define a 10 × 36 grid in the 2π image FOV, with *θ* ranging from 0° to 90° with 10° intervals and *φ* ranging from 0° to 350° with 10° intervals.
(2) Place a point source at the intersection of the grid and measure the projection point by point to generate a coarse-grid SM. The size is 360 (image domain: 10 × 36) × 1280 (projection domain: 16 × 16 × 5).
(3) Perform spline interpolation to generate a fine-grid SM with a 1° interval. As a result, the size of the fine-grid SM is 32,760 (image domain: 91 × 360) × 1280 (projection domain: 16 × 16 × 5).

The SM of the gamma imager is dependent on the energy of the radiation source. Therefore, the calibration process is repeated for each source. In this study, we chose two representative radiation sources, ^99m^Tc and ^137^Cs, to investigate the fast calibration approach. The ^99m^Tc source represents low-energy radiation and is widely used in medical applications. The ^137^Cs source has medium gamma energy and is regularly used in industrial applications.

In a typical calibration process, each of the ^99m^Tc or ^137^Cs radiation sources used in the calibration has an activity of ~15 mCi and is placed ~0.9 m away from the detector. At each measurement point, we acquire the projection in 15 s durations. The overall calibration measurement requires ~1.4 h for each imager and each radiation source. After spline interpolation, the total number of acquired photon events in each detector bin varies from 1.60 × 10^6^ to 2.17 × 10^8^ for ^99m^Tc in a 112–168 keV energy window and 5.41 × 10^6^ to 3.59 × 10^7^ for ^137^Cs in a 530–785 keV energy window.

**Figure 4:**
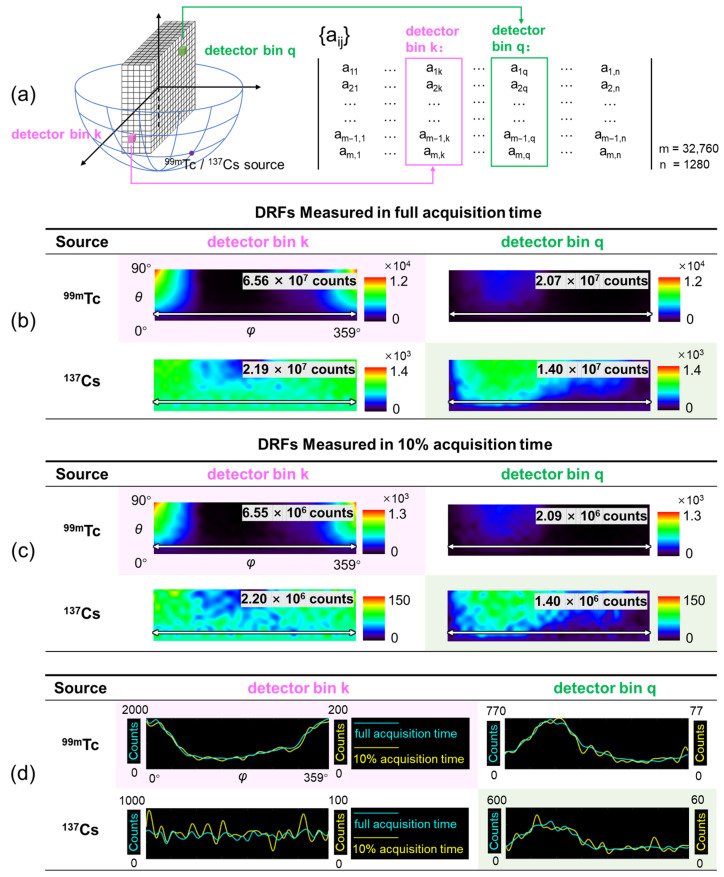
(**a**) Illustration of the geometrical relationship between calibration measurement (left) and the measured SM (right). (**b**) DRF images of two representative detector bins for ^99m^Tc and ^137^Cs sources (measured with the full acquisition time). (**c**) DRF images of two representative detector bins for ^99m^Tc and ^137^Cs sources (measured with a 10% acquisition time). (**d**) Line profiles of DRF images measured with the full acquisition time (with scale marked on the left) and 10% acquisition time (with scale marked on the right).

Figure 4a indicates the geometric relationship between the calibration measurement and the measured SM. The projection of each detector bin can be extracted as a column of SM. Figure 4b shows representative columns of the SMs measured in a typical acquisition time (~1.4 h) for two detector bins. There are 360 (*φ*) × 91 (*θ*) = 32,760 elements in each column, which is visualized as a 360 × 91 color-scale image. In this work, we define such an image as a detector response function (DRF), as it represents the detection probability distribution for a certain detector bin over all image pixels.

For a single detector bin, gamma photons emitted from different directions have various detection probabilities because of different attenuation distances between sources and the detector bin induced by other detector bins along the path. The total acquired counts in each detector bin and for each radiation source are marked in the upper-right corner of each DRF image in Figure 4b.

In Figure 4c, we show four DRFs that were also acquired for the same detector bins but with a 10% acquisition time. The counts are marked in the upper-right corner. Figure 4d demonstrates line profiles along the white double-headed arrows in Figure 4b,c for DRFs measured in full acquisition time and in 10% acquisition time. Obviously, DRFs measured with a 10% acquisition time differ from their long-time measured counterparts in terms of significantly increased statistical noise. Therefore, an effective denoising process is mandatory for the noisy SM.

In what follows, we chose DRF as the input of neural networks because (1) SM is the combination of DRFs for all the detector bins; thus, denoised SM can be given by assembling all the denoised DRFs together. (2) DRF can be shaped into a 2D smooth image with an appropriate size while retaining the physical meaning, which is beneficial for denoising.

### 2.3. Self-Adaptive, Sensitivity-Dependent Data-Grouping Strategy

**Figure 5:**
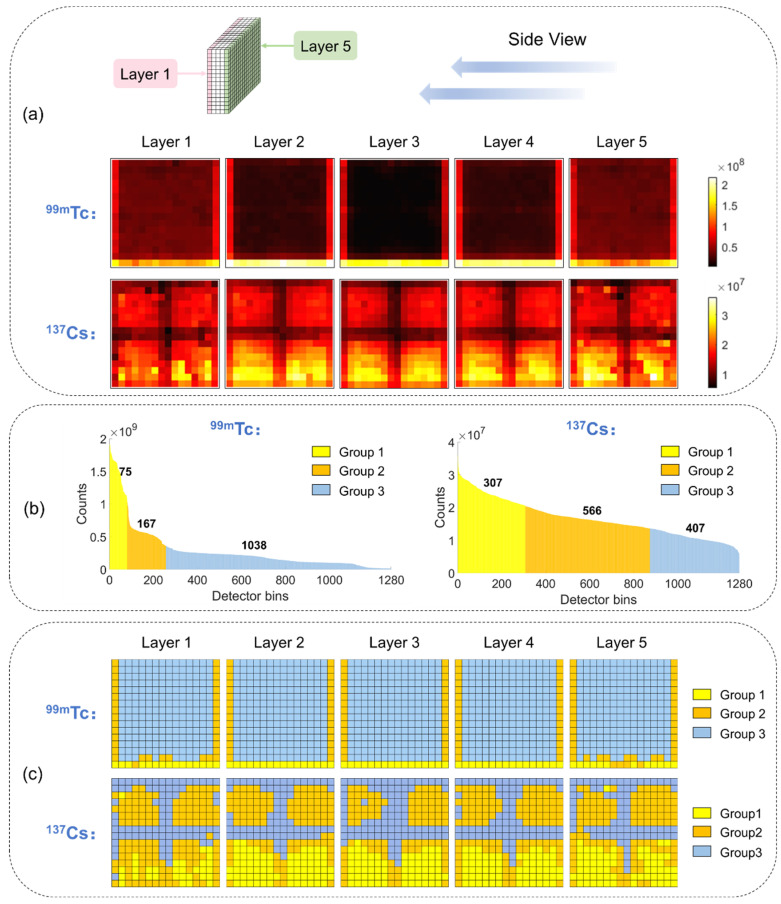
(**a**) Sensitivity maps of detector bins for ^99m^Tc and ^137^Cs sources. (**b**) Histogram of total counts of DRFs for each detector bin and grouping results for ^99m^Tc and ^137^Cs sources. (**c**) The position maps of detectors in each data group for ^99m^Tc and ^137^Cs sources.

Figure 4b,c shows prominent sensitivity variation (expressed as the sum of values in each DRF image) across different detector bins. In Figure 5a, we show sensitivity maps over the entire detector block for ^99m^Tc and ^137^Cs sources. The maps are displayed from the side view, and layers 1 to 5 are indicated in the upper row in Figure 5a. Clearly, the sensitivity of each detector bin varies with the position of the detector bin as well as the source type. For the ^99m^Tc source, the detector bins on the surface have more counts due to the low penetrating capability of the 140 keV gamma ray. The last row of detector bins for each layer has the highest sensitivity due to the lower-hemisphere FOV. Additionally, the discrepancy of properties between each crystal bar and each electronic signal read-out element contributes to the non-uniformity. For the ^137^Cs source, the “cross” pattern is caused by the signal read-out setting described in Section 2.1. Since the signals in each sub-block are read out individually, if a photon interacts with the detector block through Compton scattering and deposits a portion of energy in two sub-blocks, the produced signals on one sub-block or both sub-blocks may be low enough to be rejected by the energy window discrimination logic, leading to a low photon event distribution on the edge of each sub-block. Since it has been shown that the optimal parameter set of a denoising network is highly relevant to the count level of the images [37], the non-uniformity of sensitivity poses a challenge in training denoising networks.

To address this issue, we separated the DRF images into multiple groups according to each detector bin’s sensitivity. For each group, the denoising network training and parameter optimization processes were performed individually.

To accommodate the system response discrepancy for different radioactive sources and different machines, we implemented a self-adaptive, unsupervised K-means clustering algorithm in data grouping. The Euclidean distance was used as a metric of similarity for detector bins’ sensitivity (i.e., the sum of counts in each DRF image). Cluster centroids were initialized with random items, and then DRFs of 1280 detector bins were automatically categorized into three groups according to their sensitivity.

In Figure 5b, we show the total counts in each detector bin in descending order, and the grouping result is labeled with different colors. In both cases of the ^99m^Tc and ^137^Cs sources, we defined three data groups. For the ^99m^Tc source, there are 75 DRFs in Group 1 with an average count level of 1.6853 × 10^8^, 167 DRFs in Group 2 with an average count level of 6.9547 × 10^7^, and 1038 DRFs in Group 3 with an average count level of 2.1096 × 10^7^. For the ^137^Cs source, Group 1 has 307 DRFs with 2.4511 × 10^7^ counts on average, Group 2 has 566 DRFs with 1.6439 × 10^7^ counts on average, and Group 3 has 407 DRFs with 1.0682 × 10^7^ counts on average.

We further show the position maps of detectors in each data group (labeled in different colors) for the ^99m^Tc source and ^137^Cs source in Figure 5c. For each radioactive source and each DRF group, an individual network is trained.

### 2.4. Deep-Learning-Based Denoising

We proposed two deep learning networks for the SM denoising task: a U-net encoder-decoder network [39] and a residual U-net (Res-U-net) framework, which is the combination of a U-net and a residual connection [42]. Both networks accept DRF images as the network input and produce denoised DRF images as the output.

#### 2.4.1. Network Architectures

##### U-Net Architecture

**Figure 6:**
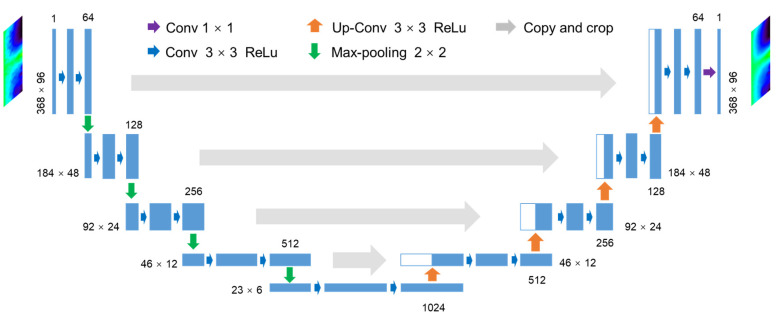
Architecture of the U-net network.

Figure 6 illustrates the U-net network architecture in this study. The width of each block indicates the number of feature maps in the layer, the length denotes the input size of the matrix, and the arrows stand for different operations. The entire network consists of an encoder, a bottleneck, and a symmetrical decoder, making up a U-shape. The encoder contains four stacks; in each stack, there are 2 convolutional layers with a 3 × 3 kernel followed by a rectified linear unit (ReLU), and a 2 × 2 max pooling layer with a stride of 2. The bottleneck has 2 convolutional layers. The decoder consists of four stacks of convolutional layers and up-convolutional layers which expand the feature maps. A fully convolutional layer is added to the end to match the feature maps to the label. Between the encoding layers and their corresponding decoding layers, there are skip connections to propagate low-level features to high-resolution layers and compensate for information loss in max pooling.

We created two adaptions from the original U-net [39]. First, image padding was applied in each convolutional layer to keep a constant size of feature maps. Second, since there are 4 max pooling layers and 4 up-convolutional layers, we adapted the length and width of the input image to be multiples of 16 so that the output image had the same size as the input.

##### Res-U-Net Architecture

**Figure 7:**
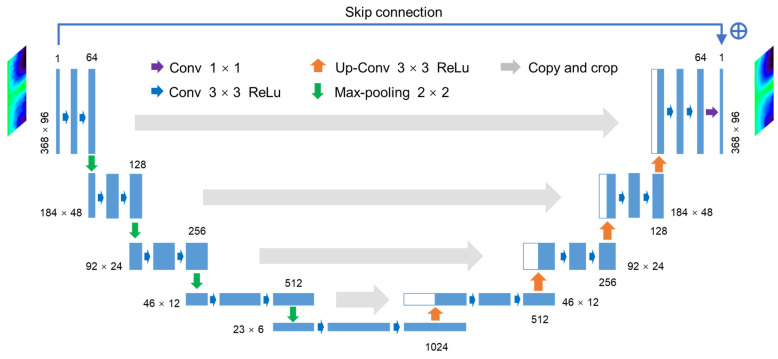
Architecture of the Res-U-net network.

Different from the U-net network, in the Res-U-net network structure (as shown in Figure 7), a skip connection is added between the input and output of the whole network. The adoption of the residual connection concept could ease the training of the network, resolve the degradation problem, and potentially improve training accuracy.

#### 2.4.2. Dataset Preparation and Network Training

The training and testing datasets were produced from the SM calibration measurements described in Section 2.2 with the following steps:

(1) By using all the events acquired in the full acquisition time of Imager 1, we produced a full-count SM (FC-SM).
(2) We generated low-count SM (LC-SM) by randomly picking 10% events from the fully acquired list mode data, representing an SM that can be measured with a 10% acquisition time.
(3) We extracted 1280 pairs of full-count DRFs and low-count DRFs from FC-SM and LC-SM and used them as the label and input dataset, respectively, which were fed into the deep networks. For each source energy and each DRF group, an individual network was trained.
(4) We repeated down-sampling steps (2) and (3) 20 times to produce 20 independent LC-SMs and used all of them as the training data so that the deep networks had sufficient input data to avoid overfitting.

One should note that (1) to match the magnitude of FC-SM, each LC-SM is multiplied by 10, and (2) due to 4 pairs of max pooling and up-convolutional layers in both U-net and Res-U-net architectures, the length and width of input matrix are preferably multiples of 16 to match the size of output figures with that of the input DRFs. Therefore, we added paddings around the input DRFs and transformed their size into 368 × 96.

We ran the network training process separately for each gamma energy and each of the data groups, as described in Section 2.3. All the training data were extracted from the calibration measurements for Imager 1.

We evaluated the efficacy of the trained denoising network with two approaches:

***Intra-device testing.*** We produced another 10 LC-SMs with measured data of Imager 1 as the testing data (statistically independent of the training data). The denoised SMs were compared to the FC-SMs.

***Inter-device testing.*** We produced 1 FC-SM and 10 LC-SMs from the calibrated data of Imager 2. Instead of training other denoising networks for Imager 2, we directly used the network trained with data of Imager 1 to denoise Imager 2′s LC-SMs. We expected this approach to reveal the potential of real-world acceleration of the calibration process in a volume production pipeline since long-time calibration measurement is required for only one device.

However, when implementing inter-device evaluation, the count levels of Imager 1 and Imager 2 were different, even for DRFs from the same detector bin, leading to a mismatch between the training and testing data noise levels. This was caused by the different properties of scintillation crystals and digital processing units from different devices. On the other hand, due to the non-linear response of the networks, the mismatch of the count level should be taken care of. Therefore, we applied detector-by-detector scaling to compensate for the mismatch as follows:

(1) Calculate DRF-wise scaling factors $\left\{F_{j}\right\}$, $F_{j}=\frac{total\;counts\;of\;the\;j^{th}\;DRF\;from\;Imager1}{total\;counts\;of\;the\;j^{th}\;DRF\;from\;Imager2}$;
(2) Generate the input DRFs: $DRF_{j}^{input}=DRF_{j}^{Imager2}\times F_{j}$;
(3) Apply the denoising networks on $\left\{DRF_{j}^{input}\right\}$ and obtain the outputs $\left\{DRF_{j}^{output}\right\}$;
(4) Implement inverse scaling on the outputs and obtain $\left\{DRF_{j}^{final}\right\}$: $DRF_{j}^{final}=DRF_{j}^{output}÷F_{j}$;
(5) Re-organize $\left\{DRF_{j}^{final}\right\}$ to form a denoised SM of Imager 2.

#### 2.4.3. Implementation Details

We used a batch size of 16 in all the training tasks. The epoch numbers of each network were empirically chosen to assure convergence, as listed in Table 1. In all the cases, we used the MSE loss function, the Adam optimizer, with an initial learning rate of 0.0001 and an exponential decay rate of 0.996.

All the computations were carried out on a workstation equipped with an NVIDIA GeForce RTX 2080 GPU card. We used a hybrid programming framework with MATLAB V9.8 and Python V3.6 with the PyTorch framework.

### 2.5. Conventional Gaussian-Filtering-Based Denoise Approach

**Figure 8:**
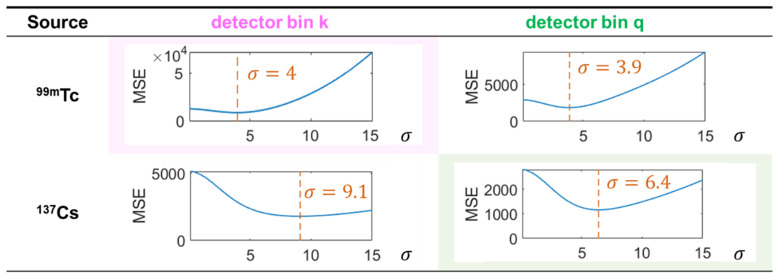
σ-MSE curves of representative detector bins for ^99m^Tc and ^137^Cs sources.

We also implemented a traditional Gaussian-filtering-based denoising method for comparison. For each individual DRF image, we filtered the image with a 2D Gaussian filtering kernel as follows:

$$G\left(x,y\right)=\frac{1}{2\pi \sigma^{2}}e^{-\frac{x^{2}+y^{2}}{2\sigma^{2}}}$$

where *σ* denotes the standard deviation of the Gaussian kernel function. To obtain the best Gaussian filtering performance for fair comparisons, we tested on the LC-SM (Imager 1) data (multiplied by 10 to match the magnitude of FS-SM (Imager 1)) with σ ranging from 0.1 to 15 pixels with a step size of 0.1 pixels for each detector bin. We used a figure-of-merit of the mean square error (MSE) between the full-count DRF image and the filtered low-count DRF image to determine an optimal σ for each DRF. Figure 8 illustrates the MSE curves of two representative detector bins (as indicated in Figure 4a) for ^99m^Tc and ^137^Cs sources. The optimal σ values that yielded the smallest MSE are marked in each sub-figure.

### 2.6. Performance Evaluation

#### 2.6.1. SSIM between System Matrices

The structural similarity index (SSIM) directly reflects the difference between FC-SM and the denoised LC-SM. SSIM between two system matrices is represented by the mean SSIM value of each corresponding DRF; additionally, SSIM for each DRF group is calculated by the mean. For two system matrices composed of *N* DRFs, the formula can be written as follows:

$$SSIM\left(SM_{1},SM_{2}\right)=\frac{\sum_{i=1}^{N}SSIM\left(DRF_{i}\_SM_{1},DRF_{i}\_SM_{2}\right)}{N}$$

#### 2.6.2. Positioning Bias

**Figure 9:**
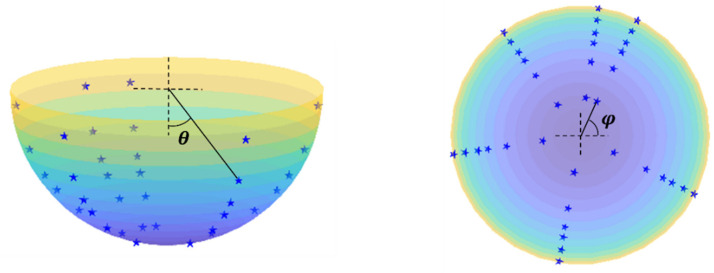
Distribution map of 36 different testing positions.

We tested the positioning accuracy by imaging a single point source at exactly known angular positions. We experimentally placed a point source at 6 × 6 different positions with *θ* = {17°, 35°, 46°, 53°, 64°, 81°} and *φ* = {64°, 82°, 127°, 189°, 261°, 333°}. The distribution map of testing positions is shown in Figure 9. At each point, we collected around 1M photon events. Each reconstructed image was calculated with 10,000 MLEM iterations. The experiments were conducted twice, one with a ^99m^Tc source and the other with a ^137^Cs source.

We calculated the positioning bias, which refers to the deviation between the reconstructed position of the radioactive source and the ground truth (denoted as $\theta_{true}$ and $\varphi_{true}$). The reconstructed position $\hat{\theta }$ and $\hat{\varphi }$ is determined by the centroid of the image in both *θ* and *φ* directions:

$$\hat{\theta }=\frac{\sum_{\theta }\sum_{\varphi }\theta \times v\left(\theta ,\varphi \right)}{\sum_{\theta }\sum_{\varphi }v\left(\theta ,\varphi \right)}$$

$$\hat{\varphi }=\frac{\sum_{\varphi }\sum_{\theta }\varphi \times v\left(\theta ,\varphi \right)}{\sum_{\varphi }\sum_{\theta }v\left(\theta ,\varphi \right)}$$

where v(θ,φ) represents the value at pixel location (θ,φ) of the reconstructed image. Then, positioning bias was calculated by:

$$bias=\arccos\left(\frac{\vec{\left(\hat{\theta },\hat{\varphi }\right)}\cdot \vec{\left(\theta_{true},\varphi_{true}\right)}}{\left|\vec{\left(\hat{\theta },\hat{\varphi }\right)}\right|\times \left|\vec{\left(\theta_{true},\varphi_{true}\right)}\right|}\right)$$

#### 2.6.3. FWHM Resolution

Image resolution is also an important image quality index. We calculated the full-width-half-maximum (FWHM) resolution from the reconstructed single-point-source images described in Section 2.6.2. We fit each image with a 2D non-isotropic Gaussian function, from which we calculated the FWHM of the point source in both *θ* and *φ* directions. Then, we calculated the FWHM resolution as

$$resolution=\sqrt{{\left(FWHM_{\theta }\right)}^{2}+{\left(FWHM_{\varphi }\right)}^{2}}$$

## 3. Results

### 3.1. Intra-Device Evaluation

#### 3.1.1. Denoised SMs

**Figure 10:**
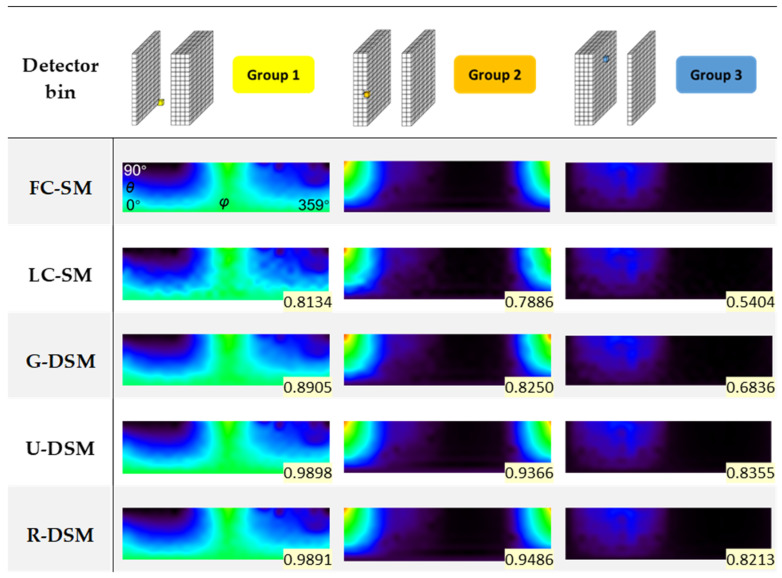
Representative DRF images of FC-SM, LC-SM, G-DSM, U-DSM, and R-DSM for ^99m^Tc source. SSIM value between each DRF image and FC-SM case is given in the bottom-right corner.

**Figure 11:**
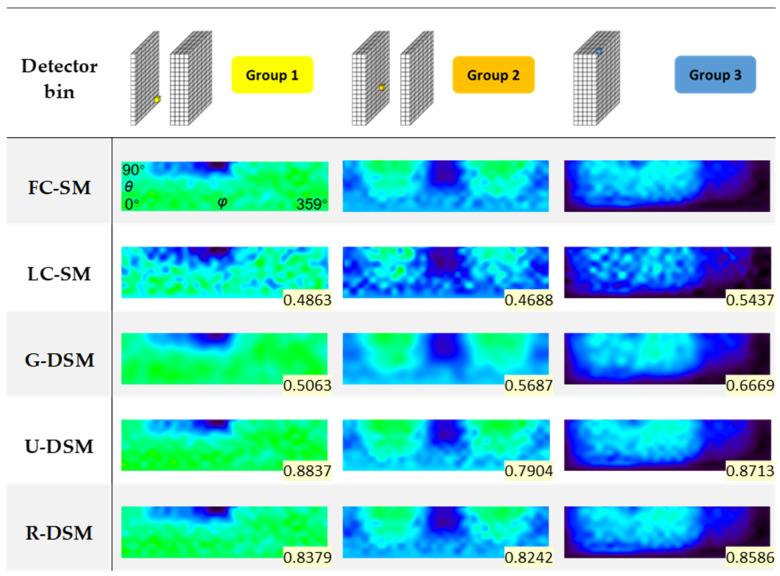
Representative DRF images of FC-SM, LC-SM, G-DSM, U-DSM, and R-DSM for ^137^Cs source. SSIM value between each DRF image and FC-SM case is given in the bottom-right corner.

We chose 10 LC-SMs of Imager 1 as the testing set to perform intra-device evaluation. Figure 10 and Figure 11 show representative DRF images of FC-SM, LC-SM, Gaussian-filtering-based denoised SM (G-DSM), U-net-based denoised SM (U-DSM), and Res-U-net-based denoised SM (R-DSM) for ^99m^Tc and ^137^Cs sources, respectively. We also statistically calculated the SSIM value between each DRF image and the corresponding FC-SM case, shown in the bottom-right corners of the images. For each group, we chose one representative detector bin (indicated in the first row in Figure 10 and Figure 11 with a highlighted box) and plotted its DRFs in the rest of the rows.

For both the ^99m^Tc source and ^137^Cs source, the DRFs of LC-SMs (third row in Figure 10 and Figure 11) are evidently different from those of FC-SMs (second row) due to increased noise. Compared with LC-SMs, the DRFs of G-DSMs (fourth row) are much smoother after Gaussian filtering but with an unavoidable loss of details. DRFs of U-DSMs (fifth row) and those of R-DSMs (sixth row) are visually more similar to those of FC-SMs after U-net-based denoising and Res-U-net-based denoising, respectively. R-DSMs yield slightly better recovery of details.

The mean and standard deviation (SD) of SSIM calculated for 10 testing LC-SMs and FC-SM, as well as those between DSMs and FC-SM for ^99m^Tc and ^137^Cs sources, are listed in Table 2 and Table 3, respectively. For both ^99m^Tc and ^137^Cs sources, the three denoising methods can improve SSIM, among which U-DSMs and R-DSMs reach higher SSIM values, while G-DSMs have the worst performance.

#### 3.1.2. Performance of Reconstructed Images—Positioning Bias

In terms of image reconstruction evaluation, we tested the 36 different point source positions described in Section 2.6.2. The projections used for reconstruction were also measured in experiments with a count level of 1M. Each reconstructed image was obtained using the MLEM algorithm with 10,000 iterations.

**Figure 12:**
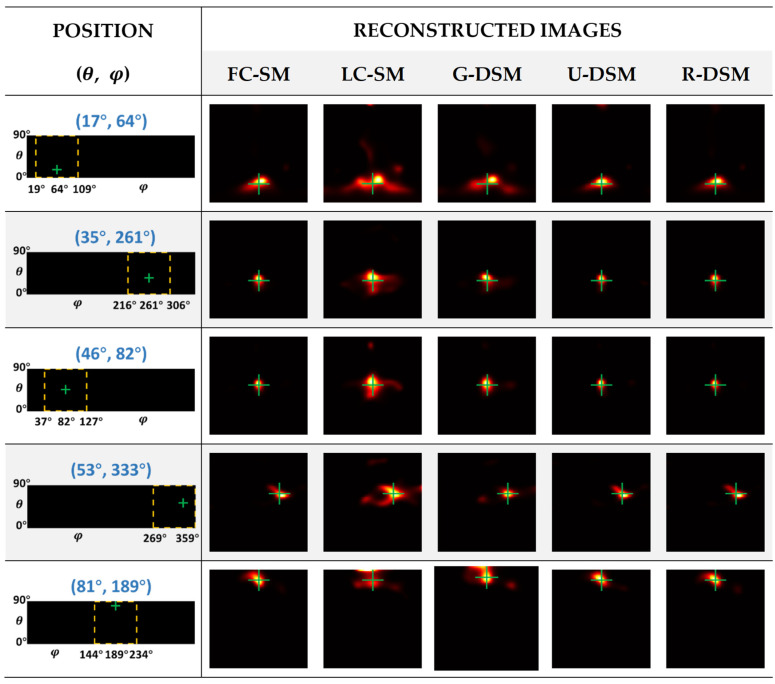
Reconstructed images of different SMs at representative positions for ^99m^Tc source in intra-device evaluation.

**Figure 13:**
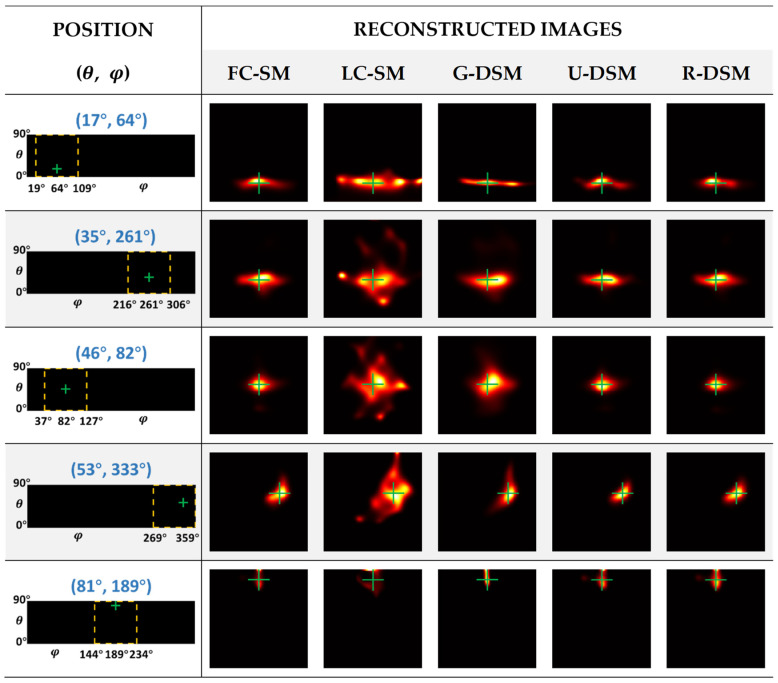
Reconstructed images of different SMs at representative positions for ^137^Cs source in intra-device evaluation.

The reconstructed images of ^99m^Tc and ^137^Cs point sources at five representative positions are illustrated in Figure 12 and Figure 13, respectively. The yellow box and green cross in each of the images in the first column indicate the zone for displaying the zoomed images and the true position of the point source. One can observe in Figure 12 and Figure 13 that the image quality of LC-SM is poor with dispersive hot-dot artifacts. Using Gaussian-filtered SMs moderately improves the image quality; however, in certain positions, there are still visible artifacts, which lead to notable positioning bias. After implementing U-net-based denoising and Res-U-net-based denoising, the reconstructed images were obviously more similar to those with FC-SM, leading to better positional accuracy.

**Figure 14:**
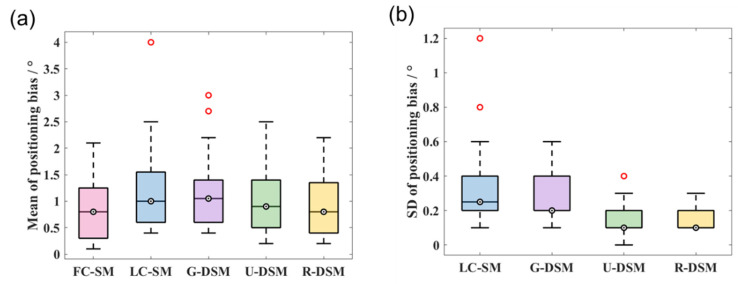
Box plots of (**a**) mean values and (**b**) SD values of positioning bias at 36 different source positions for ^99m^Tc source in intra-device evaluation.

**Figure 15:**
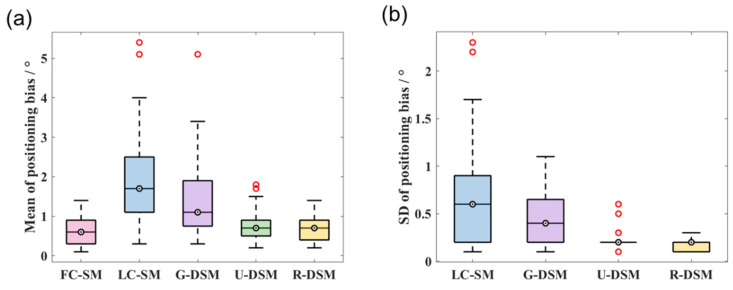
Box plots of (**a**) mean values and (**b**) SD values of positioning bias at 36 different source positions for ^137^Cs source in intra-device evaluation.

In Figure 14 and Figure 15, we present box plots for the mean value and SD of the positioning bias of reconstructed images at the 36 different source positions indicated in Figure 9. We first calculated the mean value and SD for the 10 testing datasets at each source position for LC-SM, G-DSM, U-DSM, and R-DSM cases. Then, the mean and SD results at all 36 source positions were presented in box plots. It is important to note that since there is only one dataset for FC-SM, the mean positioning bias is exactly the value of the single dataset, and there is no SD statistics result for FC-SM. Both U-net and Res-U-net-based denoising achieve <2.5° positioning bias for ^99m^Tc source and <2° for ^137^Cs source, outperforming LC-SM and G-DSM. Additionally, U-DSM and R-DSM have a lower bias SD, indicating that the deep-learning-based denoising methods are more robust for different LC-SMs than Gaussian filtering. In Figure 15b, the bias SD of U-DSM concentrates around 0.2°, making the box plot a line.

#### 3.1.3. Image Performance—FWHM Resolution

**Figure 16:**
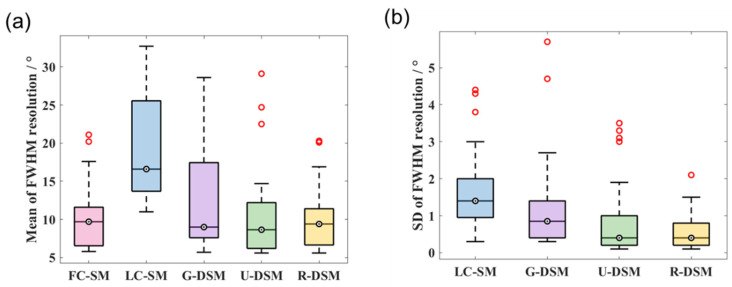
Box plots of (**a**) mean values and (**b**) SD values of FWHM resolution at 36 different source positions for ^99m^Tc source in intra-device evaluation.

**Figure 17:**
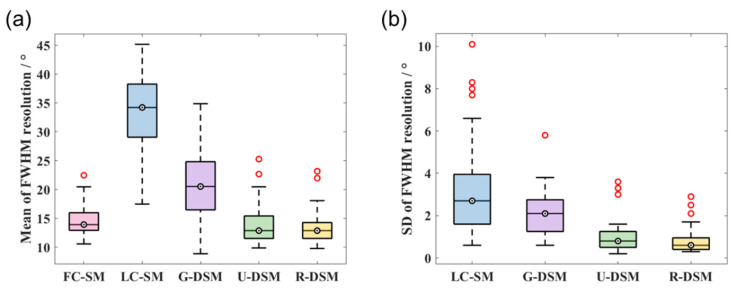
Box plots of (**a**) mean values and (**b**) SD values of FWHM resolution at 36 different source positions for ^137^Cs source in intra-device evaluation.

The mean and SD values of FWHM resolution for ^99m^Tc and ^137^Cs at 36 different source positions are shown in Figure 16 and Figure 17, respectively. For the ^99m^Tc source, the mean resolution for FC-SM, U-DSM, and R-DSM mostly stayed below 20°, better than LC-SM and G-DSM. For the ^137^Cs source, both U-net- and Res-U-net-based deep learning methods achieved around 10~20° image resolution with a few exceptions, outperforming the 20~45° resolution with LC-SM and 10~35° with G-DSM. The SD values of U-DSM and R-DSM were also much lower for both ^99m^Tc and ^137^Cs sources, indicating that the SMs with deep learning denoising methods yield more robust image reconstruction.

### 3.2. Inter-Device Evaluation

#### 3.2.1. Imaging Performance—Positioning Bias

**Figure 18:**
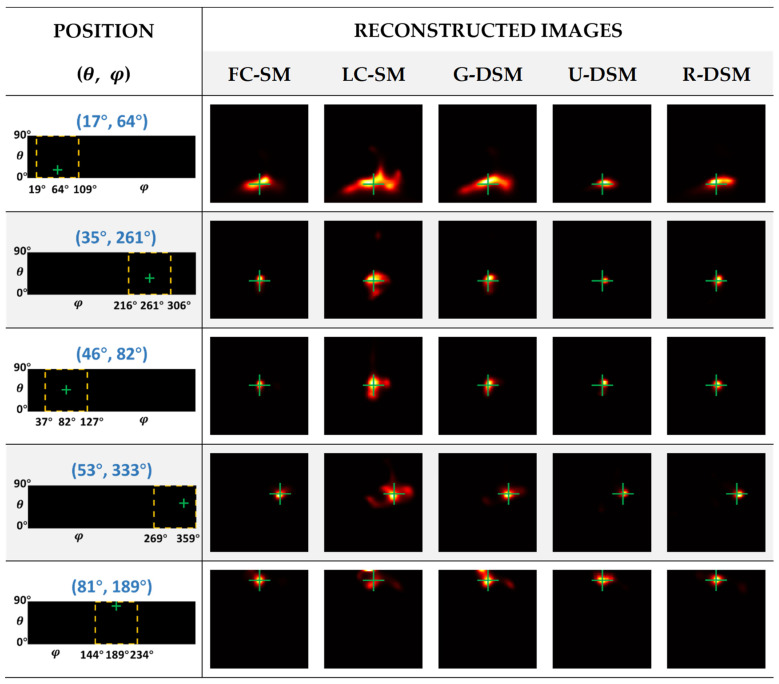
Reconstructed images of different SMs at representative positions for ^99m^Tc source in inter-device evaluation.

**Figure 19:**
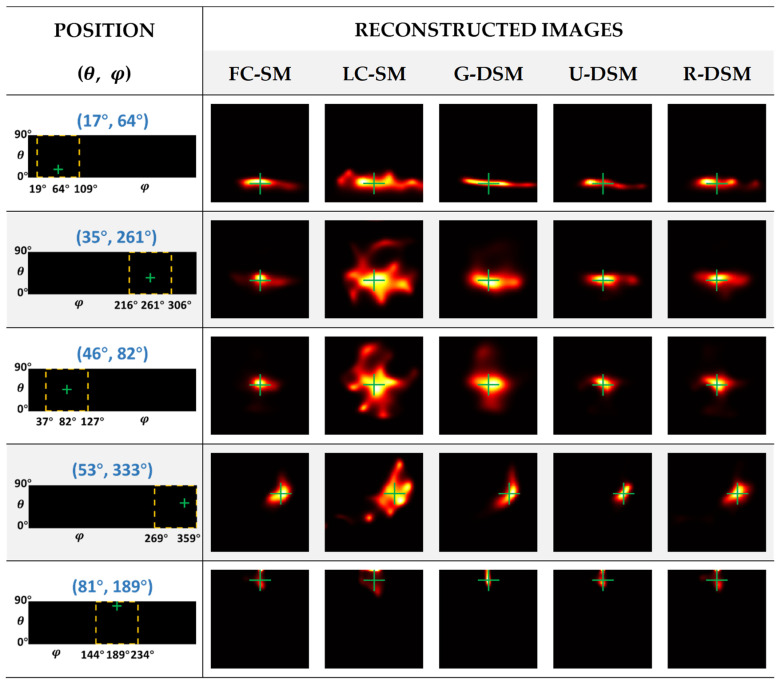
Reconstructed images of different SMs at representative positions for ^137^Cs source in inter-device evaluation.

As described in Section 2.4.2, for inter-device evaluation, we trained the denoising networks with data measured in Imager 1 and applied the networks in the denoising tasks for Imager 2. Figure 18 and Figure 19 show the reconstructed images of ^99m^Tc and ^137^Cs point sources at five representative positions. The images in the second to sixth columns correspond to the reconstructed images using an FC-SM, an LC-SM, and three denoised SMs with the Gaussian filtering method (G-DSM), with the U-net-based denoising method (U-DSM), and with the Res-U-net based denoising method (R-DSM). One can observe severe distortion with a noisy LC-SM (third column) or G-DSM (fourth column). U-DSM and R-DSM (fifth and sixth columns) yield better image quality and visually more similar image shapes to the FC-SM cases (second columns).

**Figure 20:**
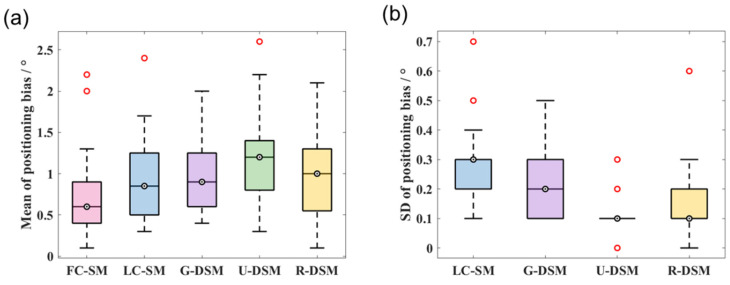
Box plots of (**a**) mean values and (**b**) SD values of positioning bias at 36 different source positions for ^99m^Tc source in inter-device evaluation.

**Figure 21:**
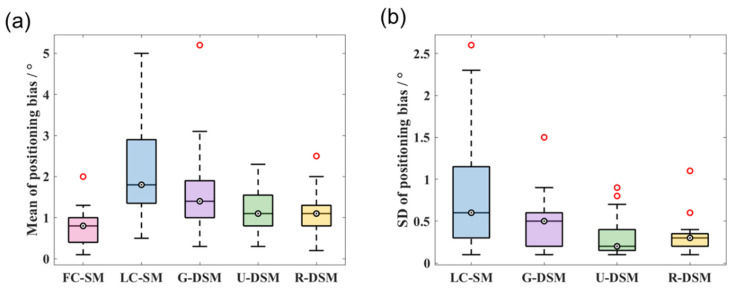
Box plots of (**a**) mean values and (**b**) SD values of positioning bias at 36 different source positions for ^137^Cs source in inter-device evaluation.

Quantitative analyses of the mean and SD values of positioning bias are summarized in Figure 20 and Figure 21 for the two radiation sources. For the ^99m^Tc source, the positioning bias is <2.6° in all cases. Although the image quality of LC-SM has an evident degradation, as shown in Figure 18, the positioning bias does not significantly increase, probably due to the centroid calculation step. In general, LC-SM, G-DSM, U-DSM, and R-DSM show comparable positioning accuracy, among which U-DSM performs slightly poorer. However, as shown in Figure 20b, the SD values of positioning bias for U-DSM and R-DSM are significantly smaller than those for other cases. For the ^137^Cs source (Figure 21a), U-DSM and R-DSM achieve <2.5° average positioning bias, close to that of FC-SM. For LC-SM and G-DSM cases, the mean positioning bias is higher, ranging up to 5°. In Figure 21b, the SD values of the positioning bias for U-DSM and R-DSM cases outperform those for LC-SM and G-DSM cases.

#### 3.2.2. Imaging Performance—FWHM Resolution

**Figure 22:**
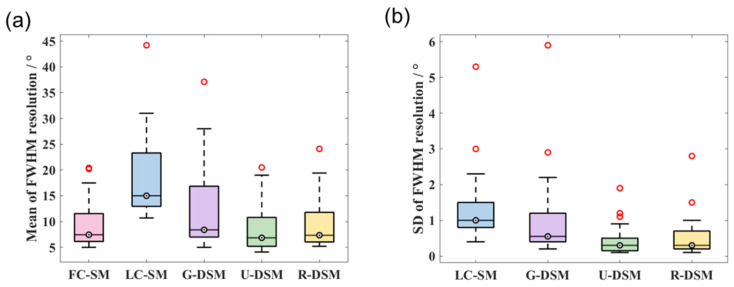
Box plots of (**a**) mean values and (**b**) SD values of FWHM resolution at 36 different source positions for ^99m^Tc source in inter-device evaluation.

**Figure 23:**
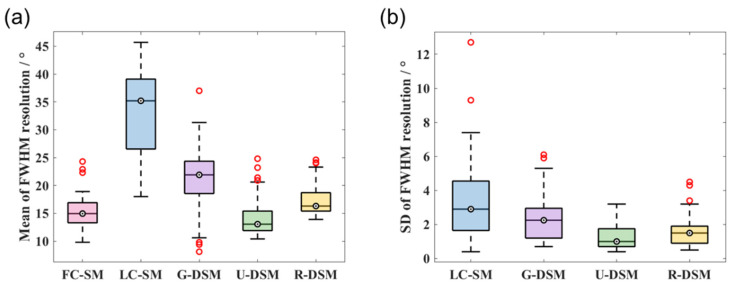
Box plots of (**a**) mean values and (**b**) SD values of FWHM resolution at 36 different source positions for ^137^Cs source in inter-device evaluation.

The FWHM resolution performance analyses are summarized in Figure 22 and Figure 23 for ^99m^Tc and ^137^Cs sources, respectively. Figure 22a and Figure 23a clearly reveal that with U-DSM and R-DSM, the mean values of image resolution are obviously better than the LC-SM and G-DSM cases and are close to the FC-SM case. The SD values of resolution (as shown in Figure 22b and Figure 23b) also demonstrate the advantage of deep-learning-based denoising over LC-SM and G-DSM cases. There are no significant differences between the imaging performance using U-net and Res-U-net networks.

## 4. Discussion

In this study, we proposed a deep-learning-based denoising method to realize time-efficient SM calibration for a 4π-view gamma detector. Two network architectures were investigated, including U-net and Res-U-net, and they both outperformed the non-denoised LC-SM and the denoised SM with conventional Gaussian filtering method in terms of more accurate source position estimation and improved FWHM resolution. The trained networks were validated with the measured data both from the same imager device as well as a different imager to test the versatility of the proposed method. With our proposed method, the system matrix calibration time can be significantly reduced from 1.4 h to 8 min while positioning accuracy and image resolution remain comparable.

To accommodate the significant response discrepancy between different detector elements, we proposed a self-adaptive data-grouping method and trained a separate network for each group. We clustered the DRF images into three different groups to accommodate different noise levels. The number of groups was chosen as a balance among various considerations, e.g., count distribution determined by signal read-out setup, image FOV, and attenuation features of gamma photons in GAGG(Ce). Using more groups might further reduce the discrepancy but at the cost of data processing complexity. Having fewer DRF images in one group may also reduce the training capacity for each network and cause over-fitting. However, the data-grouping strategy can be flexible for different system designs.

**Figure 24:**
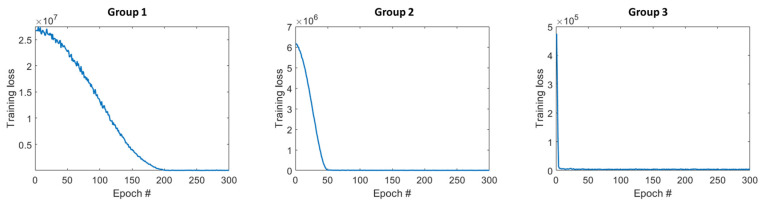
Training loss of different DRF groups with U-net model for ^99m^Tc.

**Figure 25:**
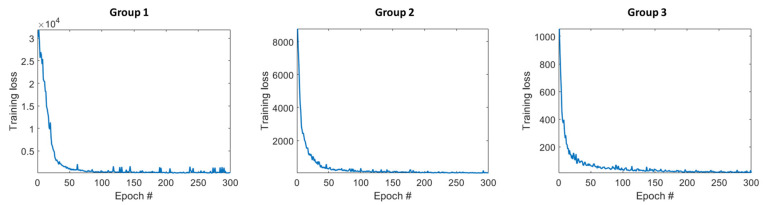
Training loss of different DRF groups with Res-U-net model for ^99m^Tc.

The convergence property and model performance varied among different DRF groups. Taking ^99m^Tc as an example, Figure 24 and Figure 25 show the training loss of three different groups for the U-net model and Res-U-net model, respectively. For both networks, Group 1 takes the largest number of epochs to converge, and Group 3 takes the fewest. This is because Group 1 comprises the fewest DRF images; thus, there are fewer iterations in one epoch, and Group 3 has more iterations in one epoch. In Table 4, we list the mean and SD of SSIM of different DRF groups calculated between 10 testing LC-SMs and FC-SM as well as those between DSMs and FC-SM for ^99m^Tc. For all the cases, SSIM decreases from Group 1 to Group 3. We believe this is because the detector bins in Group 1 have higher sensitivity and lower noise; therefore, they are more similar to the full-count case. In general, the deep-learning-based denoising approach can improve the SSIM of all three DRF groups and outperforms Gaussian filtering.

The imaging performance of ^99m^Tc and ^137^Cs sources is different. One can notice in Figure 12, Figure 13, Figure 18 and Figure 19 that the image quality of ^99m^Tc is intrinsically better than that of ^137^Cs, with fewer dispersive artifacts. Additionally, the image degradation of ^137^Cs is also much more severe than ^99m^Tc, referring to positioning accuracy as well as resolution. We believe that is because the higher-energy gamma photons of ^137^Cs (662 keV) have stronger penetration capability through the detector, which reduces the sensitivity and increases the noise. On the other hand, increased Compton scattering interactions for the ^137^Cs source may also lead to image degradation. However, compared with ^99m^Tc, the proposed deep-learning-based denoising methods have more significant improvements for ^137^Cs. We believe this is because the adverse impacts of LC-SM are stronger for ^137^Cs than for ^99m^Tc, especially when focusing on positioning bias.

There are other ways to further optimize our work. First, the performance of deep learning is highly dependent on the extensiveness of training data, so it would be better to use data generated from more devices to train the networks. In the present study, we only used the SM data from one device (Imager 1) for training. Additionally, when performing an inter-device evaluation, the mismatch of noise levels caused by different hardware leads to performance degradation compared to intra-device evaluation. In future work, we will utilize SM data from different devices for network training to improve reliability. Second, in this study, we selected two radioactive isotopes, ^99m^Tc and ^137^Cs, as representatives of the most regularly used gamma sources in medical and industrial applications. We planned to test the method with expanded collections of gamma sources in further implementations. Third, in this study, we mainly focused on applying deep learning denoising on the system matrix and practically resolving the problem of the gamma imager calibration process. Therefore, we utilized classical network architectures to primarily prove the feasibility of the approach. The hyperparameters of the networks were chosen referring to existing works which have achieved satisfactory results [35,39]. However, more comprehensive optimization of the network parameters may further improve the performance. In future work, we plan to conduct an ablation study on the network parameters (e.g., number of convolutional layers, kernel size, optimizer) for better results and explore other deep-learning models (e.g., generative adversarial network (GAN) [43]) for the SM denoising task. Additionally, the extension of datasets mentioned above may also help improve the model’s efficiency.

Our proposed deep-learning-based denoising method generally applies to other imaging systems that rely on an experimental calibration step to accommodate comprehensive system response factors in an accurately measured SM. Our proposed method effectively addresses the challenge of long calibration time, which represents a major obstruction that limits the application of experimental measurement in real practice. We expect that the presented technique can be extended to other gamma imaging devices, including industrial gamma cameras, SPECT, and PET systems.

## 5. Conclusions

In this study, we proposed a time-efficient SM calibration method with short-time measured SM and deep-learning-based denoising. To deal with sensitivity discrepancy across different detector bins, we proposed a self-adaptive, K-means clustering method to classify DRF images into multiple groups fed to independent network training processes. We investigated two denoising networks with U-net and Res-U-net architectures and compared them against a conventional Gaussian filtering method. Through intra-device and inter-device studies, we demonstrated that the denoised SMs with deep networks effectively reduce the noise-induced image degradation and faithfully yield comparable imaging performance with the long-time measured SM. Henceforth, the system matrix calibration time can be reduced from 1.4 h to 8 min. We conclude that the proposed SM denoising approach is promising and effective in enhancing the productivity of the 4π-view gamma imager, and it is also generally applicable to other imaging systems that require an experimental calibration step.

## Figures and Tables

**Table 1:** Epoch numbers for training networks.

Epoch Number	U-Net			Res-U-Net		
	Group 1	Group 2	Group 3	Group 1	Group 2	Group 3
**^99m^** **Tc**	250	225	150	250	200	135
**^137^** **Cs**	125	125	120	120	125	130

**Table 2:** SSIM (mean ± SD) with FC-SM of LC-SMs, G-DSMs, U-DSMs, and R-DSMs for ^99m^Tc.

SM	LC-SM	G-DSM	U-DSM	R-DSM
**SSIM**	0.6484 ± 0.0005	0.7433 ± 0.0004	0.8490 ± 0.0005	0.8542 ± 0.0004

**Table 3:** SSIM (mean ± SD) with FC-SM of LC-SMs, G-DSMs, U-DSMs, and R-DSMs for ^137^Cs.

SM	LC-SM	G-DSM	U-DSM	R-DSM
**SSIM**	0.5208 ± 0.0005	0.6146 ± 0.0004	0.8641 ± 0.0007	0.8542 ± 0.0004

**Table 4:** SSIM (mean ± SD) of different DRF groups for LC-SM, G-DSM, U-DSM, and R-DSM for ^99m^Tc.

SSIM	LC-SM	G-DSM	U-DSM	R-DSM
**Group 1**	0.8984 ± 0.0010	0.9450 ± 0.0008	0.9861 ± 0.0002	0.9928 ± 0.0002
**Group 2**	0.7766 ± 0.0008	0.8267 ± 0.0007	0.9261 ± 0.0006	0.9400 ± 0.0008
**Group 3**	0.6097 ± 0.0006	0.7153 ± 0.0006	0.8267 ± 0.0006	0.8303 ± 0.0005

## Data Availability

Not applicable.
